# Antimicrobial resistance trends in clinical *Escherichia coli* and *Klebsiella pneumoniae* in Ethiopia

**DOI:** 10.4102/ajlm.v13i1.2268

**Published:** 2024-03-27

**Authors:** Abera A. Kitaba, Zelalem T. Bonger, Degefu Beyene, Zeleke Ayenew, Estifanos Tsige, Tesfa Addis Kefale, Yonas Mekonnen, Dejenie S. Teklu, Elias Seyoum, Abebe A. Negeri

**Affiliations:** 1 National Clinical Bacteriology and Mycology Reference Laboratory, Ethiopian Public Health Institute, Addis Ababa, Ethiopia

**Keywords:** antimicrobial resistance, retrospective analysis, trend analysis, *Escherichia coli*, *Klebsiella pneumoniae*, Ethiopian Public Health Institute, Ethiopia

## Abstract

**Background:**

Clinicians rely on local antimicrobial resistance pattern data to guide empiric treatment for seriously ill patients when culture and antimicrobial susceptibility testing results are not immediately available.

**Objective:**

This study aimed to analyse 5-year trends in antimicrobial resistance profiles of *Escherichia coli* and *Klebsiella pneumoniae* isolates.

**Methods:**

Bacteriology reports from 2017 to 2021 at the Ethiopian Public Health Institute were analysed retrospectively. Isolates were identified using either the VITEK 2 Compact system, the BD Phoenix M50 instrument, or conventional biochemical tests. Antimicrobial susceptibility testing was conducted using either the Kirby-Bauer disk diffusion method or the VITEK 2 Compact system and BD Phoenix M50 systems available at the time of testing. The Cochran Armitage trend test was employed to test the significance of antimicrobial resistance trends over time. *P*-values less than 0.05 were considered statistically significant.

**Results:**

Of the 5382 bacteriology reports examined, 458 (9%) were on *E. coli* and 266 (5%) were on *K. pneumoniae*. Both *K. pneumoniae* (88%) and *E. coli* (65%) demonstrated high resistance to extended-spectrum cephalosporins. However, both *K. pneumoniae* (14%) and *E. coli* (5%) showed lower rates of resistance to carbapenems compared to other antimicrobials. In *K. pneumoniae*, resistance to carbapenems (from 0% to 38%; *p* < 0.001) and ciprofloxacin (from 41% to 90%; *p* < 0.001) increased significantly between 2017 and 2021.

**Conclusion:**

Both organisms showed very high resistance to broad-spectrum antibiotics. Additionally, *K. pneumoniae* demonstrated a statistically significant rise in ciprofloxacin and carbapenem resistance.

**What this study adds:**

This study emphasises the significance of regular reporting of local antimicrobial resistance patterns as this information can guide appropriate empiric therapy and efforts to address antimicrobial resistance issues.

## Introduction

The emergence and spread of antimicrobial-resistant *Enterobacteriaceae*, particularly *Escherichia coli* and *Klebsiella pneumoniae*, is a critical health problem and threatens the effective prevention and treatment of serious infections.^1^ Antimicrobial-resistant *E. coli* and *K. pneumoniae* are responsible for a high proportion of serious nosocomial infections, including urinary tract, bloodstream, abdominal, and respiratory tract infections, and are a serious concern for global health and development.^2,3^ *E. coli* and *K. pneumoniae* develop resistance to beta-lactam antibiotics primarily by producing carbapenemase and extended-spectrum beta-lactamase enzymes.^4,5,6,7^

*Klebsiella pneumoniae* tends to be significantly more resistant than *E. coli*, with carbapenem resistance rates exceeding 25% reported in several southern European countries.^7^ Infections caused by carbapenem-resistant, extended-spectrum beta-lactamase-producing, and multidrug-resistant *E. coli* and *K. pneumoniae* are more difficult to treat and occur at higher frequencies.^2,3,7^ The United States Centers for Disease Control and Prevention has classified carbapenem-resistant *E. coli* and *K. pneumoniae*, as urgent threats and extended-spectrum beta-lactamase-producing *E. coli* and *K. pneumoniae*, as serious threats, whereas the World Health Organization classified them as critical priority pathogens requiring the development of new active antimicrobial agents.^2,3^

Globally, several studies and antimicrobial resistance surveillance systems have reported increasing rates of antimicrobial resistance among *E. coli* and *K. pneumoniae.*^8,9,10,11,12,13^ For example, a 10-year retrospective study in China found that the rate of carbapenem-resistant *K. pneumoniae* increased significantly, from 6.7% in 2010 to 56.7% in 2019.^12^ Similarly, a surveillance report from a hospital in Malawi revealed a substantial increase in ciprofloxacin-resistant *E. coli* (from 0.0% in 1998 to 31.1% in 2016) and ciprofloxacin-resistant *Klebsiella* species (from 1.7% in 1998 to 70.2% in 2016).^14^ Several scientific reports out of Africa have emphasised the fast-growing threat of antimicrobial resistance.^8,11,14,15,16,17,18^ The threat appears to be receiving more attention in the region than ever before, as evidenced by an increase in the number of publications on the subject, as well as the initiatives by the African Society for Laboratory Medicine and the African Centers for Disease Control and Prevention.^19^

Even though studies in Ethiopia have reported a high burden of antimicrobial resistance among *E. coli* and *K. pneumoniae*,^8,17,18^ there is limited information on the trends of antimicrobial resistance among these pathogens in Ethiopia. Therefore, the current study was designed to analyse 5-year antimicrobial resistance trends among *E. coli* and *K. pneumoniae* recovered from specimens referred to the Ethiopian Public Health Institute. The findings of our study may provide local antimicrobial resistance patterns of *E. coli* and *K. pneumoniae* to clinicians to guide empiric treatment for seriously ill patients when culture and antimicrobial susceptibility testing results are not immediately available. Moreover, the findings may provide information for initiatives aimed at combating the problem of antimicrobial resistance, including infection prevention and control practices and antimicrobial stewardship.

## Methods

### Ethical considerations

Due to the retrospective nature of this study, we were unable to obtain consent from the patients who provided specimens. Therefore, a waiver of informed consent to conduct the study was requested and approved by the institutional review board of the Ethiopian Public Health Institute with approval number EPHI-IRB-413-2021. To maintain confidentiality, patient names and other personal identifiers were encrypted, and unique identification numbers were utilised to identify data. Therefore, the study was conducted in accordance with Helsinki Declaration as revised in 2013.

### Data collection

This retrospective study included routine bacteriology culture reports of *E. coli* and *K. pneumoniae* isolates obtained from various clinical specimens at the Ethiopian Public Health Institute between January 2017 and December 2021. The laboratory provides diagnostic services as well as a range of research activities. It was accredited by the Ethiopian Accreditation Service following the requirements of the International Organization for Standardization 15189:2012.

### Isolate identification and antimicrobial susceptibility testing

Isolation and identification of the bacteria were achieved by culturing the specimens onto appropriate culture media and incubating them at 35 °C – 37 °C following laboratory standard operating procedures. The isolated bacteria were identified using one of the following laboratory methods available at the time of testing: VITEK 2 Compact system (bioMérieux, Marcy-l’Étoile, France), BD Phoenix M50 (Becton, Dickinson and Company, Franklin Lakes, New Jersey, United States), and standard biochemical tests. For standard biochemical tests, the following biochemical tests were used along with Gram staining for isolate identification: triple sugar iron agar (Oxoid Ltd., Basingstoke, Hampshire, England), lysine iron agar (Biomark, Pune, Maharashtra, India), sulfide indole motility (HIMEDIA, Mumbai, Maharashtra, India), Simmons citrate agar (Biomark, Pune, Maharashtra, India), urea agar (HIMEDIA, Mumbai, Maharashtra, India), and oxidase (Liofilchem, Roseto degli Abruzzi, Italy). Antimicrobial susceptibility testing was carried out using either the Kirby-Bauer disk diffusion method on Muller Hinton agar (Oxoid Ltd., Basingstoke, Hampshire, England), the VITEK 2 Compact system, or the BD Phoenix M50 system. The results of antimicrobial susceptibility tests (susceptible, intermediate, and resistant) were interpreted using the latest Clinical and Laboratory Standards Institute M100 criteria available at the time of testing.^20^

Quality control for antimicrobial susceptibility testing and biochemical tests and culture was carried out as per the Clinical and Laboratory Standards Institute guidelines^20,21^, manufacturer instructions, and laboratory standard operating procedures using different American-type culture collection strains. *E. coli* American-type culture collection 25922, *Staphylococcus aureus* American-type culture collection 25923, and *Pseudomonas aeruginosa* American-type culture collection 27853 strains were used for quality control during antimicrobial susceptibility testing.

### Grouping antimicrobial agents for trend analysis

For trend analysis, we grouped related antimicrobials according to drug classes using the Clinical and Laboratory Standards Institute M02 guidelines.^21^ Antimicrobial agents were categorised as extended-spectrum cephalosporins, aminoglycosides, first- and second-generation cephalosporins, carbapenems, β-lactam-combination agents, ciprofloxacin, and trimethoprim/sulfamethoxazole. Resistance to at least one of the several agents within each antimicrobial class was used to define resistance at the antimicrobial class level. Isolates were considered extended-spectrum cephalosporin-resistant if they were resistant to at least one of the extended-spectrum cephalosporins: ceftazidime, cefepime, and ceftriaxone. Aminoglycoside-resistant isolates were those that were resistant to at least one of the following aminoglycosides: amikacin, tobramycin, and gentamycin. Strains that were resistant to amoxicillin/clavulanic acid or piperacillin/tazobactam were deemed resistant to a β-lactam-combination agent. Carbapenem resistance was defined as resistance to meropenem or imipenem. Strains resistant to cefazolin or cefuroxime were considered resistant to first- and second-generation cephalosporins.

### Data extraction

The bacteriology records from the laboratory logbook were entered into WHONET software (WHO Collaborating Centre for Surveillance of Antimicrobial Resistance; https://whonet.org/software.html). The following information was obtained: type of specimen, final culture results, identity of the organism (for positive cultures), susceptibility testing results, date of specimen collection and receipt at the laboratory, and patients’ demographics (age and gender). To avoid bias from repetitive culture, only data on the first isolate from each patient were included.

### Statistical analysis

The WHONET software was used to analyse resistance rates.^22^ For each species, the resistance rates were calculated by dividing the number of resistant isolates by the total number of isolates tested. This was calculated for each year, and the yearly trends over the 5 years were assessed. We also determined the distribution of pathogens according to the age and gender of patients, as well as specimen types and year of isolation. Statistical analysis was performed using R software,^23^ and the Cochran Armitage trend test was employed to test the statistical significance of antimicrobial resistance trends over time. *P*-values less than 0.05 were considered statistically significant.

## Results

**FIGURE 1:**
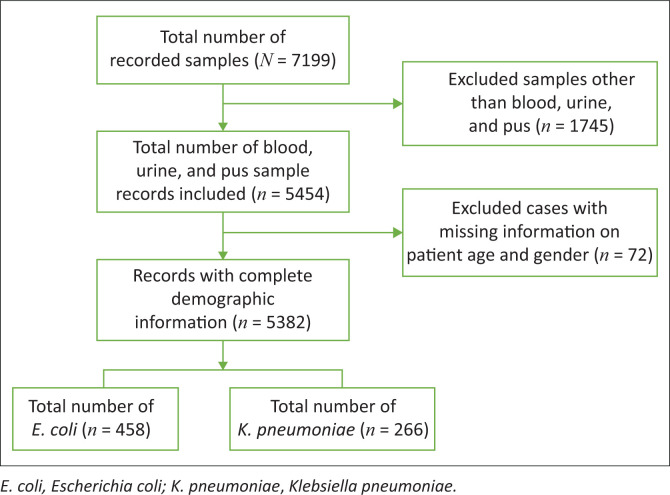
Flow diagram of applied criteria for selection of bacteriology culture data for this study. Ethiopian Public Health Institute, Addis Ababa, Ethiopia, January 2017 – December 2021.

A total of 7199 clinical specimens were collected from 2017 to 2021 (Figure 1). Specimens other than blood, urine, and pus (*n* = 1745) were excluded from this study; isolates from stool, throat swabs, nasal swabs, and other specimens were not considered pathogens. Additionally, specimens with missing information on patient age and gender (*n* = 72) were excluded from this study. In total, 5382 records of blood, pus, and urine specimens with complete information were obtained, from which 458 (9%) *E. coli* and 266 (5%) *K. pneumoniae* isolates were recovered. The combined prevalence of *E. coli* and *K. pneumoniae* was 13% (724/5382).

### Distribution of *E. coli* and *K. pneumoniae* by age, gender, and specimen type

There were more male patients (*n* = 2890; 54%) than female patients (*n* = 2492; 46%) (Table 1). The majority of the patients (*n* = 2459; 46%) were aged between 19 and 45 years. Of the 5382 specimens, 42% were urine specimens, 40% were blood samples, and 18% were pus samples. Out of the 724 isolates, 458 (63%) were *E. coli*, the majority (*n* = 356; 78%) of which were recovered from urine. The remaining 266 (37%) isolates were *K. pneumoniae*, of which 129 (50%) were recovered from blood specimens. About 82% of all *E. coli* and *K. pneumoniae* isolates were recovered from blood and urine specimens. *E. coli* (*n* = 261; 57%) was more prevalent among female patients, while *K. pneumoniae* (*n* = 153, 58%) was more common in male patients.

**TABLE 1:** Distribution of *Escherichia coli* and *Klebsiella pneumoniae* by age, gender, and specimen types. Ethiopian Public Health Institute, Addis Ababa, Ethiopia, January 2017 – December 2021.

Variable	Number of records	*E. coli*	%	*K. pneumoniae*	%
**Age in years**					
< 6	1053	19	2	124	12
6–18	479	30	6	21	4
19–45	2459	219	9	71	3
46–65	986	125	13	33	3
> 65	405	65	16	17	4
**Gender**					
Female	2492	261	10	94	4
Male	2890	197	7	153	5
**Specimen type**					
Blood	2176	18	1	129	6
Pus	954	84	9	48	5
Urine	2252	356	16	89	4

*E. coli, Escherichia coli; K. pneumoniae, Klebsiella pneumoniae*.

### Five-year resistance patterns of *E. coli* and *K. pneumoniae* isolates

**FIGURE 2:**
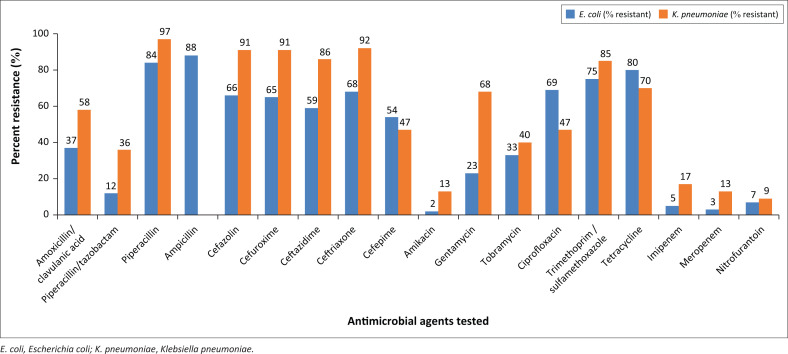
Resistance rates of *Escherichia coli* and *Klebsiella pneumoniae* to tested antimicrobials at the Ethiopian Public Health Institute, Addis Ababa, Ethiopia, January 2017 – December 2021.

Among the *E. coli* isolates, the highest rates of resistance were recorded against ampicillin (88%), piperacillin (84%), and tetracycline (80%), and the lowest resistance rates were recorded against amikacin (2%), meropenem (3%), and imipenem (5%) (Figure 2). Among the *K. pneumoniae* isolates, the highest resistance rates were observed against cefazolin (91%), cefuroxime (91%), ceftriaxone (92%), and piperacillin (97%), while the lowest resistance rates were recorded against nitrofurantoin (9%), amikacin (13%), meropenem (13%), and imipenem (17%).

### Antimicrobial resistance trends of *E. coli*

**FIGURE 3:**
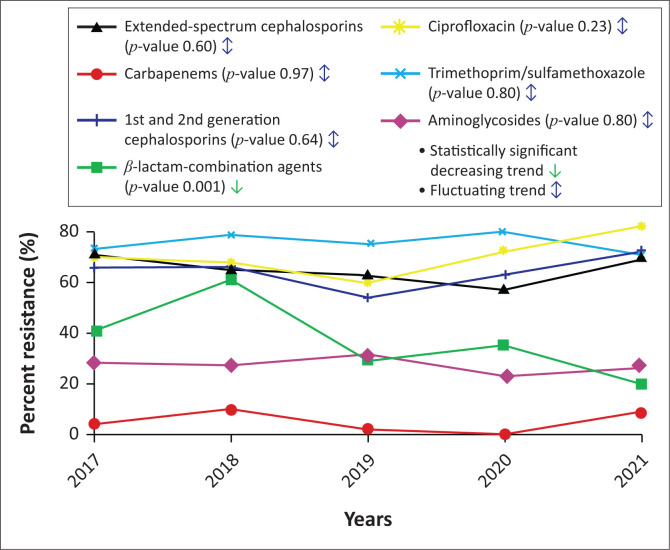
Antimicrobial resistance trends among *Escherichia coli* at the Ethiopian Public Health Institute, Addis Ababa, Ethiopia, January 2017 – December 2021.

Despite the observed high rates of resistance to common antimicrobial agents among the *E. coli* isolates in this study, we found no statistically significant increasing trends in resistance to any of the tested antimicrobials (Figure 3). The resistance rates of *E. coli* to ciprofloxacin ranged from 60% to 82% and from 71% to 80% for trimethoprim/sulfamethoxazole between 2017 and 2021. The proportion of ciprofloxacin-resistant *E. coli* was 70% in 2017 but fell to 68% in 2018 and 60% in 2019, before rising to 72% in 2020 and 80% in 2021.

Carbapenem resistance trends in *E. coli* fluctuated during the study period. In 2017, the proportion of carbapenem-resistant *E. coli* was 4%. This proportion rose to 10% in 2018, then fell to 2% in 2019 and 0% in 2020 before rising again to 9% in 2021. Resistance rates to the extended-spectrum cephalosporins (range: 57% – 71%) and first- and second-generation cephalosporins (range: 54% – 72%) were continuously high throughout the study period. In 2017, the proportion of extended-spectrum cephalosporin-resistant *E. coli* was 71%. This proportion dropped slightly to 65% in 2018, 63% in 2019, and 57% in 2020 before rising again to 69% in 2021. For the aminoglycosides, the proportion of resistant *E. coli* isolates ranged from 23% to 31% between 2017 and 2021, while for the β-lactam combinations, the proportion of resistant *E. coli* isolates dropped from 41% to 20%.

### Antimicrobial resistance trends of *K. pneumoniae*

**FIGURE 4:**
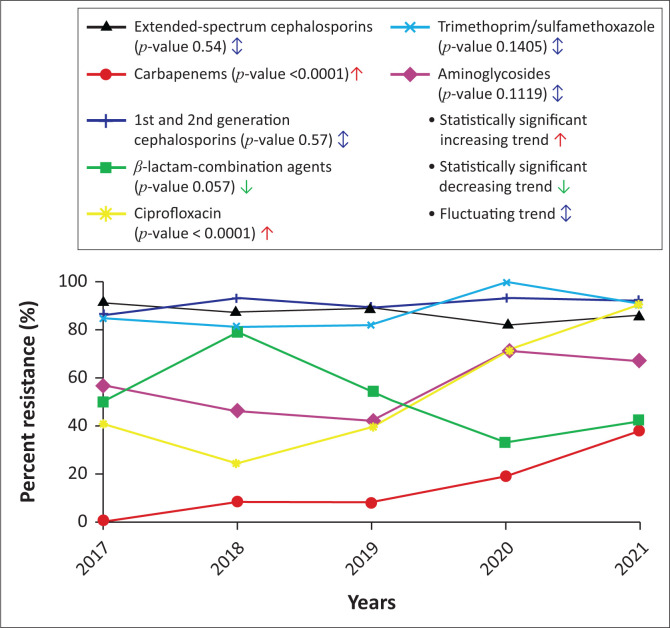
Antimicrobial resistance trends among *Klebsiella pneumoniae* at the Ethiopian Public Health Institute, Addis Ababa, Ethiopia, January 2017 – December 2021.

Overall, K. *pneumoniae* exhibited high levels of antimicrobial resistance, with 88% of the isolates resistant to extended-spectrum cephalosporins, 51% resistant to aminoglycosides, 44% resistant to ciprofloxacin, and 14% resistant to carbapenems (Figure 4). There was a statistically significant increase in ciprofloxacin resistance and carbapenem resistance rates among the *K. pneumoniae* isolates (*p* < 0.0001). Over the 5 years, the proportion of carbapenem-resistant *K. pneumoniae* increased significantly from 0% in 2017 to 8% in 2018 and 2019, and from 19% in 2020 to 39% in 2021 (*p* < 0.001). From 2017 to 2018, the proportion of ciprofloxacin-resistant *K. pneumoniae* decreased from 41% to 24%. However, by 2019, this proportion increased to 39%, before surging to 71% in 2020 and 90% in 2021 (*p* < 0.001). Over the first 3 years of the study period, the prevalence of trimethoprim/sulfamethoxazole-resistant *K. pneumoniae* ranged between 81% and 85%. However, this proportion rose to 100% in 2020 before decreasing to 91% in 2021.

Among the *K. pneumoniae* isolates, resistance rates to the extended-spectrum cephalosporins remained above 80% throughout the study period. In 2017, 57% of *K. pneumoniae* isolates were resistant to aminoglycosides. However, the resistance rate decreased to 46% in 2018 and 42% in 2019, before rising to 71% in 2020 and 67% in 2021. The proportion of *K. pneumoniae* isolates that were resistant to the β-lactam combinations ranged from 33% to 79% between 2017 and 2021. In the first 2 years of the study period, the proportion of *K. pneumoniae* isolates resistant to the β-lactam combinations increased from 50% to 79%, before decreasing to 54% in 2019. Subsequently, in both 2020 and 2021, the resistance levels dropped below 50%, reaching 33% in 2020 and 42% in 2021.

## Discussion

In this study, *E. coli* displayed high rates of resistance against ampicillin, piperacillin, and tetracycline, and low rates of resistance against amikacin and meropenem. *K. pneumoniae* exhibited high rates of resistance to cefazolin, cefuroxime, ceftazidime, trimethoprim-sulphamethoxazole, ceftriaxone, and piperacillin, and low rates of resistance against amikacin and meropenem. Regarding the trends in resistance observed over 5 years, our findings indicate that there were no statistically significant increases in resistance to the tested antimicrobials among *E. coli* isolates. However, we did observe a statistically significant rise in the rates of ciprofloxacin and carbapenem resistance among the *K. pneumoniae* isolates.

The Ethiopian Standard Treatment Guidelines for general hospitals provide comprehensive recommendations on the use of various antibiotics for empiric treatment.^24^ These guidelines consider the anatomical location and severity of different infections. For instance, in the case of sepsis, the guidelines recommend a combination of ampicillin and gentamicin, penicillin G, or a combination of an aminoglycoside or ciprofloxacin with ceftazidime.^24^ The guidelines suggest various medications, such as amoxicillin, cephalexin, ciprofloxacin, nitrofurantoin, ampicillin, gentamicin, trimethoprim-sulphamethoxazole, cefuroxime, ceftriaxone, and additional options, for the treatment of urinary tract infections.^24^ In the case of wound infections, the guidelines recommend piperacillin-tazobactam, ampicillin-sulbactam, cefazolin, amoxicillin-clavulanate, doxycycline, trimethoprim-sulphamethoxazole, and cefuroxime. These guidelines aim to ensure the effective and responsible use of antibiotics in Ethiopian hospitals.^24^

Regarding local prescribing practices, a study conducted in 2021 at Dilchora Referral Hospital in Ethiopia revealed that ciprofloxacin was the most frequently prescribed antibiotic for treating patients with urinary tract infections, followed by norfloxacin and amoxicillin/clavulanic acid.^25^ Another study at Ambo University Referral Hospital, Ethiopia, from 2019, reported that ceftriaxone was the most commonly prescribed single antibiotic, accounting for 21.7% of prescriptions.^26^ In that study, the combination of ceftriaxone and azithromycin was the most popular choice for empiric treatment of community-acquired pneumonia, accounting for 50.7% of prescriptions. The authors also highlighted a non-adherence rate of 36.4% to the national guidelines for antibiotic use. Furthermore, a study conducted at four governmental hospitals in eastern Ethiopia in 2017 identified amoxicillin, ceftriaxone, and ciprofloxacin as the top three prescribed antibacterial drugs.^27^ Lastly, research from Addis Ababa, Ethiopia, from 2016, revealed that amoxicillin was the most frequently prescribed antibiotic, accounting for 44.8% of prescriptions, followed by ciprofloxacin at 13.6%, and trimethoprim-sulphamethoxazole at 11.2%.^28^ The observed high resistance patterns of both *E. coli* and *K. pneumoniae* isolates to commonly used antibiotics may be attributed to these factors. Given that these pathogens have demonstrated high resistance to the antibiotics recommended in the Ethiopian Standard Treatment Guidelines, it is necessary to regularly revise the guidelines in accordance with local antimicrobial resistance levels.

In this study, we observed a significant increase in carbapenem resistance rates among *K. pneumoniae*, reaching 38% by the end of 2021. In agreement with our findings, China’s antimicrobial surveillance network reported a significant rise in the prevalence of meropenem-resistant *K. pneumoniae* from 2.9% in 2005 to 26.3% in 2018 (*p* < 0.001).^29^ Furthermore, a 20-year (1997–2016) report from the SENTRY Antimicrobial Surveillance Program revealed a significant increase in carbapenem resistance among *K. pneumoniae*, rising from 0.7% in 1997 to 14.2% in 2016 in Europe (*p* < 0.001).^30^ This increase in carbapenem resistance might be explained by poor hospital infection control and prevention practices and inappropriate prescription practices.^31^ Inappropriate prescription practices, in turn, may be related to the scarcity of bacteriology laboratories capable of promptly detecting resistant bacteria and providing antibiograms to clinicians in health facilities in developing countries.^31^ In contrast to our findings, however, earlier studies at 14 New York City hospitals reported a decline in carbapenem-resistant *K. pneumoniae* at 6 of the 14 hospitals (carbapenem-resistant *K. pneumoniae* decreased from 38% in 2006 to 29% in 2009; *p* < 0.001).^32^ Another study from the United States reported that the occurrence of carbapenem-resistant *K. pneumoniae* isolates declined significantly in a public health system in New York, United States, from 2016 to 2020, but increased between January 2021 and June 2022.^33^ These differences in observed trends could be attributed to strong infection control and prevention efforts done to decrease and prevent carbapenem-resistant *K. pneumoniae* infections in both hospital and community settings in New York City.^32^

Ciprofloxacin resistance rates increased significantly among *K. pneumoniae* from 2017 (41%) to 2021 (90%), consistent with findings of a study conducted in Sichuan, China, from 2017 to 2020 that reported increasing resistance of *K. pneumoniae* to ciprofloxacin from 14.7% in 2017 to 26.5% in 2020.^34^ Similarly, the SENTRY Antimicrobial Surveillance Program reported increasing rates of ciprofloxacin resistance among *K. pneumoniae* from 7.3% in 1997 to 27.9% in 2016 in Europe.^30^ Furthermore, the Taiwan Surveillance of Antimicrobial Resistance programme (2002–2012)^35^ reported a significant decrease in the proportion of ciprofloxacin-susceptible *K. pneumoniae* (average of 89.9% from 2002 to 2006 to an average of 81.6% from 2008 to 2012). Conversely, however, the Korean Antimicrobial Resistance Monitoring System reported an almost constant trend for ciprofloxacin-resistant *K. pneumoniae* from 2013 to 2015.^13^ Geographical variations may explain these observed differences.

High resistance rates to both first-line and last-resort antibiotics were observed in our study among *E. coli* and *K. pneumoniae* isolates. Overall, the rates of resistance to trimethoprim/sulfamethoxazole (*E. coli* 75% and *K. pneumoniae* 85%), ciprofloxacin (*E. coli* 69% and *K. pneumoniae* 44%), the first-generation to fourth-generation cephalosporins (*E. coli* 65% and *K. pneumoniae* 88%), and aminoglycosides (*E. coli* 28% and *K. pneumoniae* 51%) among both species during the 5-year study period were very high. This is consistent with the findings of previous studies in Ethiopia,^8,17,18^ Kenya,^36^ and Uganda.^37^ These high rates of resistance could be related to the high prevalence of irrational antibiotic use and self-prescription practices in Ethiopia.^38,39,40,41,42^ As a result, these antimicrobials may no longer be considered effective treatment options for infections caused by *E. coli* and *K. pneumoniae*. This, in turn, underlines the importance of developing bacteriology laboratory capacity in healthcare facilities to ensure that isolate identification and antimicrobial susceptibility testing results are made available for clinicians. Furthermore, antimicrobial stewardship programmes are required to monitor and regulate antibiotic use. Our findings are inconsistent with those of a retrospective analysis of data from a national surveillance network in Switzerland over 8 years (2009–2016) that showed lower resistance among *E. coli* and *K. pneumoniae* to commonly used antibiotics such as third- and fourth-generation cephalosporins (*E. coli* < 6%, *K. pneumoniae* < 5%), ciprofloxacin (*E. coli* > 14%, *K. pneumoniae* > 12%), and trimethoprim/sulfamethoxazole (*E. coli* < 23%, *K. pneumoniae* < 11%).^43^ This may be explained by the low use of antibiotics in both community and hospital settings in Switzerland.^43^

### Limitations

The lack of molecular identification for *K. pneumoniae* could have led to the misidentification of various species within the *K. pneumoniae* species complex. Due to the retrospective nature of this study, we were unable to confirm resistance genes using polymerase chain reaction. This would have provided valuable additional information regarding the presence or absence of specific resistance genes in the organisms studied, enhanced our understanding of the mechanisms underlying antibiotic resistance, and potentially allowed a more comprehensive analysis of the genetic determinants contributing to resistance patterns.

### Conclusion

Based on the findings of this study, both *K. pneumoniae* and *E. coli* showed very high resistance to both first-line and last-resort antibiotics recommended in the Ethiopian Standard Treatment Guidelines. However, the antimicrobial resistance trends for the majority of the antimicrobial agents fluctuated throughout the study period. Furthermore, *K. pneumoniae* showed a statistically significant increasing trend of resistance to carbapenems and ciprofloxacin. This is concerning, since it may compromise the treatment of critically ill patients. This necessitates continued infection control efforts, together with diagnostic and antimicrobial stewardship programmes in healthcare facilities. Additionally, it is necessary to regularly revise the guidelines in accordance with local antimicrobial resistance levels.
